# Trends in Antimicrobial Resistance of *Acinetobacter baumannii* and *Pseudomonas aeruginosa* from Bloodstream Infections: An Eight-Year Study in a Romanian Tertiary Hospital

**DOI:** 10.3390/ph18070948

**Published:** 2025-06-24

**Authors:** Alina Maria Borcan, Elena Rotaru, Laura Georgiana Caravia, Mihai-Cezar Filipescu, Mădălina Simoiu

**Affiliations:** 1 Faculty of Medicine, The University of Medicine and Pharmacy “Carol Davila”, 050474 Bucharest, Romania; alina.borcan@umfcd.ro (A.M.B.); laura.caravia@umfcd.ro (L.G.C.); madalina.simoiu@umfcd.ro (M.S.); 2 The National Institute of Infectious Diseases “Prof. Dr. Matei Bals”, 021205 Bucharest, Romania; rotaru.hellen@gmail.com

**Keywords:** antimicrobial resistance, *Pseudomonas aeruginosa*, *Acinetobacter baumannii*, bloodstream infections, healthcare-associated infections

## Abstract

**Background**: Bloodstream infections (BSIs) caused by multidrug-resistant non-fermenting Gram-negative bacilli, particularly *Pseudomonas aeruginosa* and *Acinetobacter baumannii*, represent a growing public health concern, especially in tertiary care settings. This study aimed to describe the epidemiological and antimicrobial resistance trends of *P. aeruginosa* and *A. baumannii* isolated from blood cultures over an eight-year period (2017–2024) at a tertiary infectious disease hospital in Bucharest, Romania, especially in the context of the disruption caused by the SARS-CoV-2 pandemic. **Methods**: A retrospective study was conducted on 43,951 blood cultures processed at the National Institute of Infectious Diseases. Species identification and antibiotic susceptibility testing (AST) were performed using VITEK2, MALDI-TOF MS, and supplementary phenotypic methods. AST interpretation followed EUCAST guidelines. **Results**: Out of all of the positive blood cultures, 112 (3.63%) were *P. aeruginosa* and 158 (5.12%) *A. baumannii*. Multidrug-resistance (MDR) was identified in 46% of *P. aeruginosa* and 90.73% of *A. baumannii* isolates. Resistance trends varied, with *P. aeruginosa* showing a decrease in MDR rates post-COVID-19 pandemic and following antimicrobial stewardship implementation. In contrast, *A. baumannii* displayed persistently high resistance, with carbapenem and aminoglycoside resistance rates reaching 100% by 2024. Colistin resistance, though low overall, increased in the latter years. **Conclusions**: The findings highlight the dynamic nature of antimicrobial resistance among *P. aeruginosa* and *A. baumannii*. Effective infection control and antimicrobial stewardship programs are crucial in curbing the rise of MDR strains, particularly amid healthcare system disruptions such as the COVID-19 pandemic.

## 1. Introduction

According to the World Health Organization (WHO), antimicrobial resistance (AMR) is currently the most urgent threat to global health. It is estimated that bacterial AMR was directly responsible for 1.27 million deaths in 2019 and contributed to another 4.95 million deaths. Thus, AMR should be considered a severe problem for healthcare workers worldwide [1]. In the United States alone, AMR pathogens caused more than 2.8 million infections and over 35,000 deaths annually from 2012 through 2017, according to the Center for Disease Control and Prevention (CDC) “Antibiotic Resistance Threats in the United States in 2019” report [2]. The emergence and dissemination of MDR/XDR/PDR has placed an increasing burden upon healthcare facilities across the world. From a One Health point of view and excluding healthcare-associated sources of antimicrobial resistance, dairy waste and environmental contamination contributes to an ever-growing problem [3,4]. In addition to causing death and disability, antimicrobial resistance (AMR) also has significant economic costs. The World Bank estimates that, by 2050, AMR could lead to an additional US$ 1 trillion in healthcare costs; by 2030, it could result in Gross Domestic Product losses ranging anywhere from 1 trillion US dollars to 3.4 trillion US dollars per year [1].

*Pseudomonas aeruginosa* (*P. aeruginosa*) strains have become a public health concern. *P. aeruginosa* causes severe infections, particularly in healthcare settings and immunocompromised patients. The current lack of therapeutic alternatives means that infections caused by these antibiotic-resistant bacteria pose a considerable threat regarding morbidity and mortality worldwide. The lack of adequate therapy on these infections is most definitely worrying [5].

*Pseudomonas aeruginosa* is the third most common Gram-negative species isolated from blood cultures. Estimated mortality rates vary widely, ranging from 21% to 62% [6,7,8,9,10,11,12]. It is challenging to accurately estimate the worldwide impact of *P. aeruginosa* bacteremia due to the complexities involved in mortality calculations. These complexities are influenced by the high occurrence of *P. aeruginosa* infections in critically ill patients and by the characteristics of the infecting strain, such as antimicrobial resistance and exotoxin profile. *P. aeruginosa*, including strains expressing clinically significant exotoxins encoded by ExoS and ExoU, is primarily found in moist environmental niches such as lakes, swimming pools, faucets, and sinks rather than in the human host [13,14]. The CDC has identified multidrug-resistant *P. aeruginosa* as a severe threat and a species of critical concern by the WHO. This means that this pathogen poses the highest threat to public health due to limited treatment options, a very high disease burden (high mortality and morbidity), and a marked increase in antimicrobial resistance with few or no promising antibiotics in the development stage, as of current moment. Infections with this particular pathogen are difficult to prevent and highly transmissible amongst critically ill patients. However, preventive efforts have led to a 29% decrease in infection rates since 2013, highlighting a positive trend that may continue if such measures are maintained in place and/or enhanced [15].

The CDC classified drug-resistant *Acinetobacter baumannii* (*A. baumannii*) infections as an urgent threat in the 2019 and 2021–2022 reports due to high rates of antimicrobial resistance, including strains that are resistant to all available antibiotics [15,16]. In 2024, the WHO classified resistance in *A. baumannii* as a critical priority [2]. *A. baumannii* is one of the most frequent non-fermenting bacteria encountered in clinical laboratories. Even with only about 10% of the frequency of *P. aeruginosa*, it is one of the species still most often responsible for hospital-acquired infections [17]. *A. baumannii* is a clinically problematic Gram-negative species that causes bacteremia, especially in healthcare settings, with an estimated mortality risk ranging from 20 to 39% [18,19,20]. The high mortality rates in intensive care units linked to *A. baumannii* bacteremia are due to more severe comorbidities and other possible co-infections. Community-acquired *A. baumannii* bacteremia cases have a slightly lower mortality rate. *A. baumannii* infections are closely linked to healthcare-associated settings. Thus, more extended hospital stays increase the chance of *A. baumannii* bacteremia [21]. It is well-documented that hospital environments are rich sources of *A. baumannii*, found on the surfaces of portable medical equipment, mattresses, and sinks. These bacteria can colonize the skin, nasal tract, and trachea. Isolates from patients are often the same as those found in hospital environments during their stay. It is challenging to determine whether colonization from hospital environments is temporary and could lead to spreading to community reservoirs. *A. baumannii* is also found in various other places, such as soil and fertilizer. However, it still needs to be determined how relevant these habitats are in the context of community-acquired infections [22,23,24,25,26].

Although *A. baumannii* accounts for a relatively low percentage of overall bacteremia cases, multidrug resistance is globally problematic for this species [27]. The CDC classified drug-resistant *A. baumannii* infections as an urgent threat in the 2019 and 2021–2022 reports due to high rates of antimicrobial resistance, including strains that are pandrug-resistant [5,6]. In 2024, the WHO classified resistance in *A. baumannii* as a critical priority [2]. In Taiwan, over 15% of *A. baumannii* isolates are carbapenem-resistant. Globally, over 71% are multidrug-resistant. In the United States, 27% of mechanically ventilated patients were colonized with a multidrug-resistant strain of *A. baumannii* [7,21,28]. Approximately 1% of all *A. baumannii* isolates in a multicenter study were pan-drug resistant, with colistin being the only reliable antimicrobial agent against these strains [29]. Hospital-acquired infections account for 75% of *A. baumannii* infections and about 86% of antimicrobial-resistant strains [30]. Infection prevention strategies have reduced *A. baumannii* bacteremia, indicating that continued efforts could reduce the disease burden [18]. In an effort to better highlight life-threatening, antibiotic resistant, and highly virulent bacterial pathogens, both *P. aeruginosa* and *A. baumannii* have been included in the “ESKAPE” acronym. The “ESKAPE” group also contains *Enterococcus faecium*, *Staphylococcus aureus*, *Klebsiella pneumononiae*, and *Enterobacter* spp. This group of Gram-positive and Gram-negative bacteria can cause life-threatening healthcare-associated infections and are more and more resistant to commonly used antibiotics, necessitating increased epidemiological attention.

Given the lack of reliable data in Southeast Europe regarding AMR trends in the context of healthcare-associated infections with *P. aeruginosa* and *A. baumannii*, this study comes to mitigate this lack of data regarding antimicrobial resistance and incidence of such infections, especially in a pre- and post-pandemic setting, encompassing the 2017–2024 period.

## 2. Results

During the 8 years of the study, 43,951 blood cultures were received by the microbiology department, obtained from patients hospitalized in different clinical departments of the National Institute of Infectious Diseases “Prof. Dr. Matei Bals” of Bucharest. Bacterial growth was detected in 3082 samples, resulting in a positivity rate of 7.01%. In the sample, 3.63% (N = 112) were identified as *P. aeruginosa*, and 5.12% (N = 158) were identified as *A. baumannii*.

### 2.1. Characteristics of Pseudomonas aeruginosa Positive Blood Cultures (BC)

**Figure 1:**
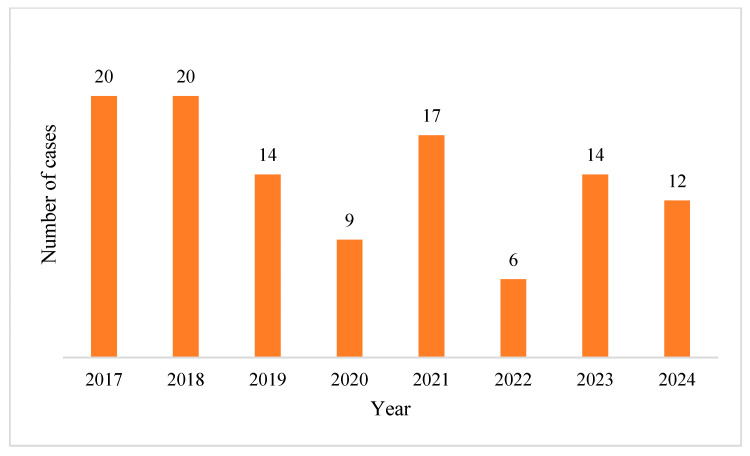
Yearly distribution of *P. aeruginosa* positive BC’s.

The highest incidence was observed in the years 2017 and 2018 (*n* = 20), while the lowest number of isolated strains was recorded in 2022 (*n* = 6). No statistically significant trend was observed over the study period (*p* = 0.118) (Figure 1).

**Figure 2:**
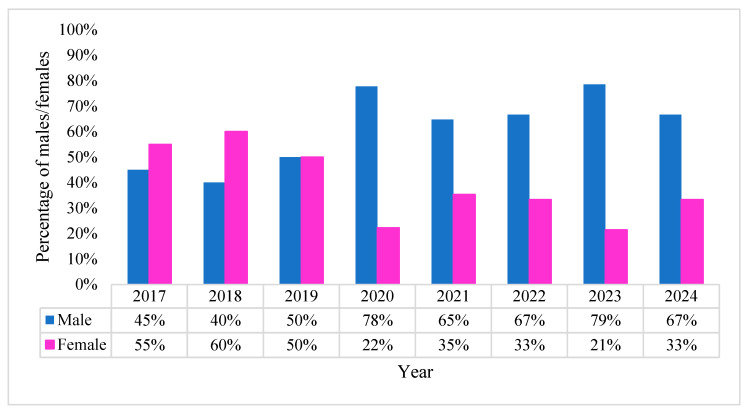
Gender distribution of *P. aeruginosa* positive BC’s.

More than half of the patients with *P. aeruginosa* bacteremia were male (57%), observing a decreasing trend among females starting in 2020 (Figure 2). It continued to decrease through 2021 and 2022 (corroborating with pandemic years) to the lowest point of 21% in 2023. The male group heavily outnumbered the female group during the heaviest years of the pandemic, with the highest peak (79%) in 2023 and maintaining the majority in 2024 (67%). Some conclusions can be extrapolated from this data. The overrepresentation of males can be attributed to the higher rate of hospitalization from SARS-CoV-2 infection amongst this group (especially in critical care settings), coupled most likely with a higher rate of severe comorbidities compared to women.

Overall, resistance to combinations of beta-lactam antibiotics and beta-lactamase inhibitors (BL/BLI) tested (piperacillin/tazobactam) was 41.18%. Piperacillin/tazobactam resistance tended to be lower than in the initial year (2017) when we observed the highest pre-pandemic incidence of 60% and the highest post-pandemic rate of 100% in the year 2023. The lowest occurrence was in 2020 (14.29%).

**Figure 3:**
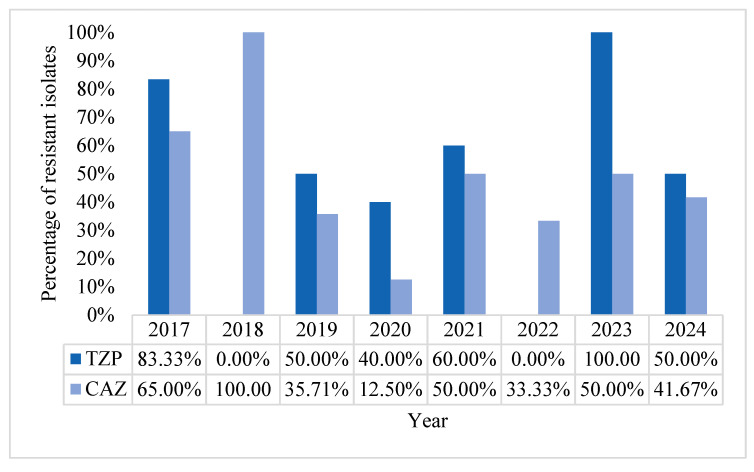
Percentage resistance rates of *Pseudomonas aeruginosa* to ceftazidime and piperacillin-tazobactam, in percentages.

General 3rd generation cephalosporin resistance during the study period was of 42.16% for ceftazidime. The lowest incidence of ceftazidime resistance was encountered in 2020, with a value of 12.50% and registering over 30% resistance in the post-pandemic period (Figure 3). The downward trend of resistance to ceftazidime in the post-pandemic years can be attributed to better antimicrobial stewardship guidelines and/or lower critical care admission numbers.

**Figure 4:**
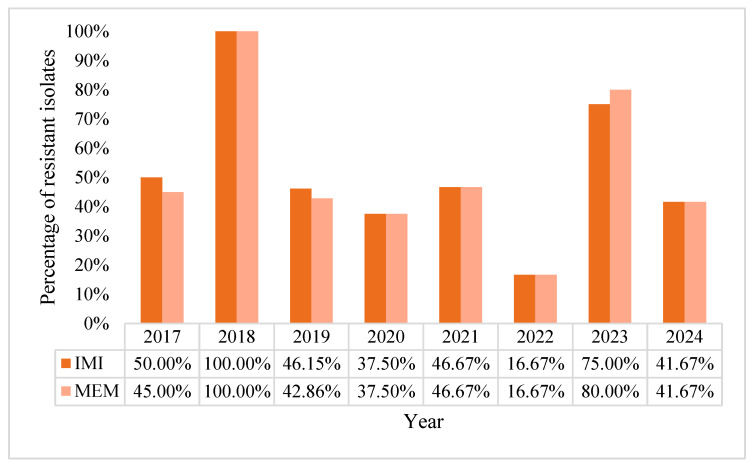
Percentage of resistance rates of *Pseudomonas aeruginosa* to imipenem and meropenem, in percentages.

Overall resistance to carbapenems was 41.84% for imipenem and of 41% for meropenem. The trend fluctuated over the years. We noticed a declining trend in the years 2021 and 2022, with the lowest registered value in 2022 (16.67%), followed by a resurgence in carbapenem-resistance in 2023 registering 75% resistance for imipenem and 80% for meropenem (Figure 4).

**Figure 5:**
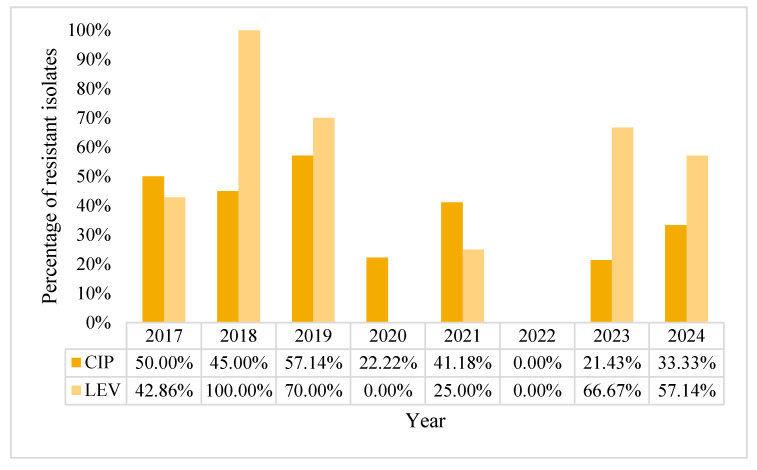
Percentage of resistance rates of *Pseudomonas aeruginosa* to fluoroquinolones, in percentages.

The overall resistance rate to fluoroquinolones was 38.39% for ciprofloxacin and 36.92% for levofloxacin. A downward trend can be noticed during the pandemic years, with 2022 being characterized by 0% for fluoroquinolones, though this data should be interpreted with caution (Figure 5). The small number of BSI with *P. aeruginosa* in that year (n = 6) might affect statistical relevance of these results. Indeed, there was somewhat of a downward trend in AMR of *P. aeruginosa* for fluoroquinolones during pandemic years, but with a worrying recrudescence to almost pre-pandemic levels in the years 2023 and 2024.

**Figure 6:**
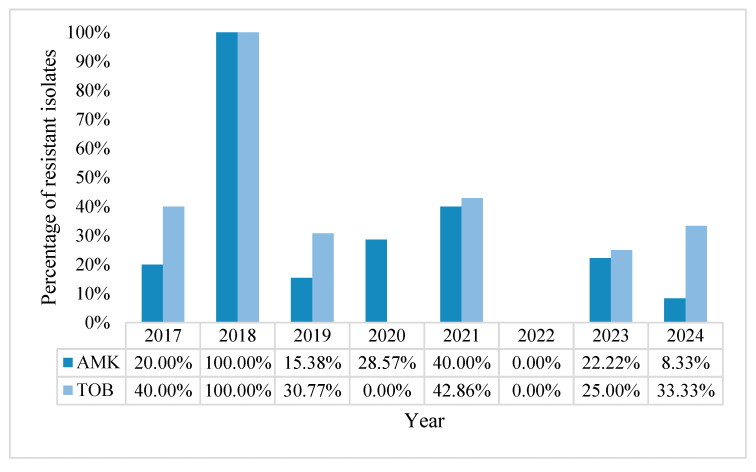
Percentage of resistance rates of *Pseudomonas aeruginosa* to aminoglycosides, in percentages.

The overall resistance rate to aminoglycosides was 21.57% for amikacin and 30.53% for tobramycin. Again, the 0% rate of resistance to aminoglycosides in the year 2022 might be due to the small sample size. The AMR rates remained somewhat low in the years following the pandemic (Figure 6).

**Figure 7:**
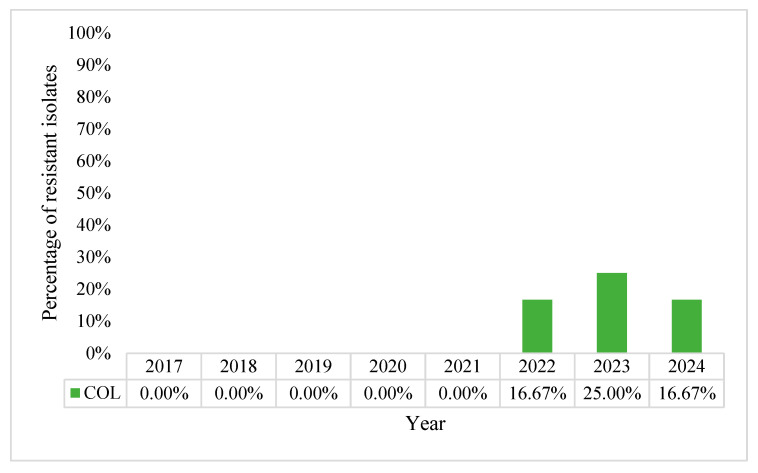
Percentage resistance rates of *Pseudomonas aeruginosa* to colistin, in percentages.

The overall resistance to colistin rate during the study was 5.00%. The highest incidence of colistin-resistant strains was observed in 2023 when it peaked at 25%, following the tendency started in 2022 when it encountered a 16.67% resistance, and maintaining the same rate for the year 2024 (Figure 7). This worrying trend of resistance to colistin highlights the dire need for new antibiotic compounds capable of treating such serious infections, especially given the fact that colistin is used as a salvage treatment for XDR Gram-negative infections. It also follows similar trends in other countries and other healthcare facilities around the world, as more and more colistin-resistant strains of *P. aeruginosa* are being isolated.

**Figure 8:**
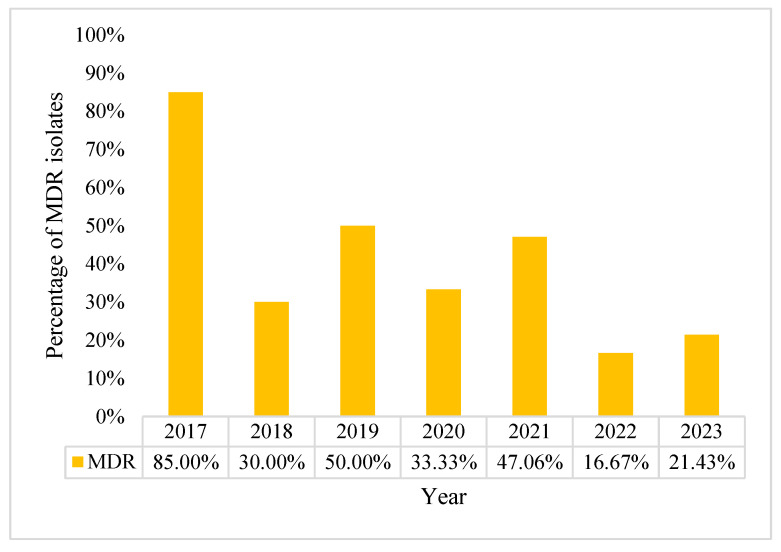
Distribution of multidrug resistant of *P. aeruginosa*.

From the overall number of MDR strains of *P. aeruginosa* (n = 46), 46% are MDR, most of which were encountered in 2017 (n = 17/20) with an incidence rate of 85%. Starting in 2021, we noted a general downward trend followed by a small increase in MDR strains, reaching 40% of all *P. aeruginosa* BSI in 2024 (Figure 8).

### 2.2. Acinetobacter baumannii Positive BCs Statistics

**Figure 9:**
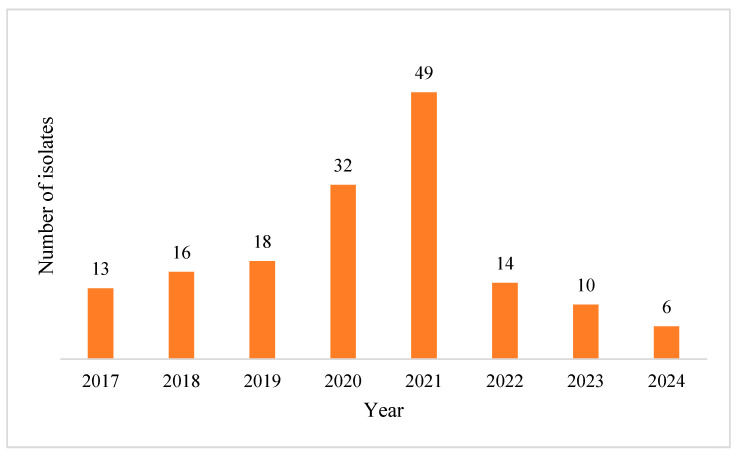
Numeric yearly distribution of *A. baumannii*-positive BCs.

Over the eight years of the study, 158 strains were isolated from positive BCs. We observed an upward trend from 2017 until the zenith (*n* = 49) encountered in 2021, followed by a downward trend over the next years (*n* = 14) in 2022, (*n* = 10) in 2023 and (*n* = 6) in 2024 (Figure 9).

**Figure 10:**
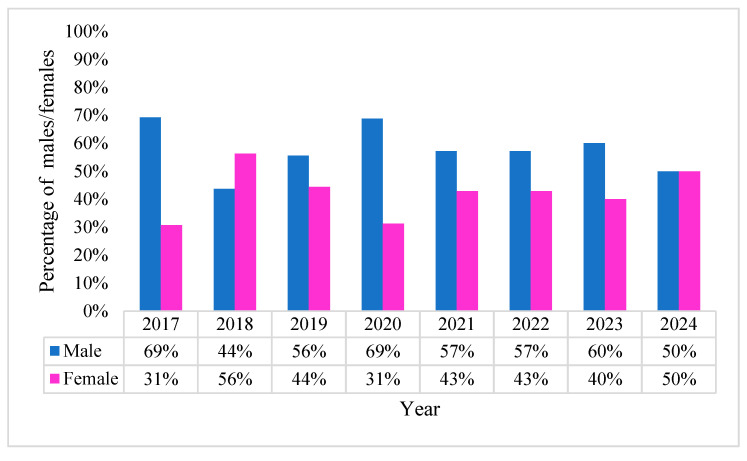
Percentage gender distribution of *A. baumannii* positive BCs.

Gender differentiation again reveals the male group’s predominance, with fluctuating rates over the years but maintaining an overall incidence rate of 58.86% compared to 41.14% for the female group (Figure 10).

**Figure 11:**
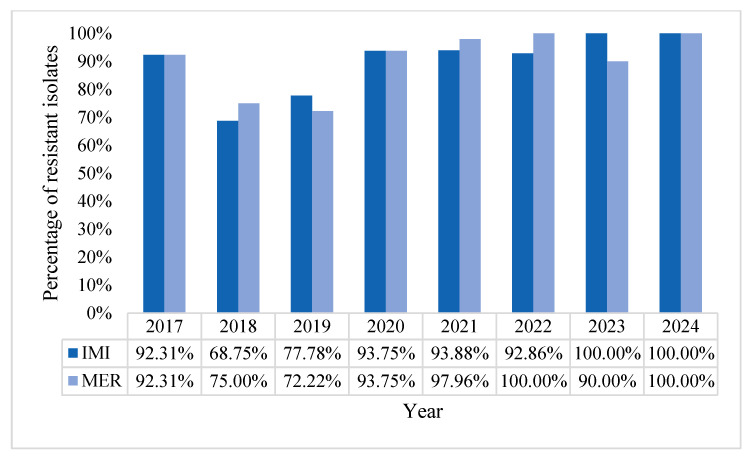
Percentage of carbapenem-resistant strains.

The overall incidence of resistance rates for carbapenems was 89.87% for imipenem and 91.14% for meropenem. Throughout the years comprised in the study, resistance to carbapenems remained at very high levels, worryingly peaking at 100% resistance rate in 2024 (Figure 11).

**Figure 12:**
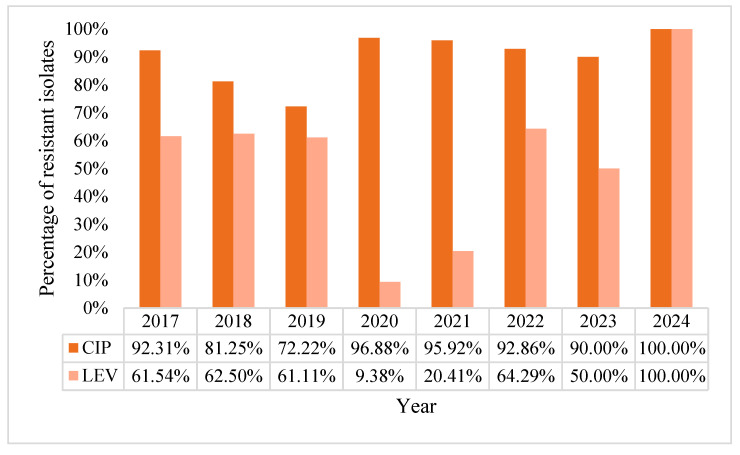
Percentage of resistance rates of *Acinetobacter baumannii* to fluoroquinolones.

The general fluoroquinolone resistance rates were 91.14% for ciprofloxacin and 39.24% for levofloxacin. The proportion of fluoroquinolone-resistant *A. baumannii* BSI strains fluctuated over the years, but maintained an upward resistance trend in post-pandemic period peaking in the case of ciprofloxacin in 2024 at 100%. For levofloxacin, the trend continuously progressed from 2020 to 2024, with a 100% resistance by 2024 (Figure 12).

**Figure 13:**
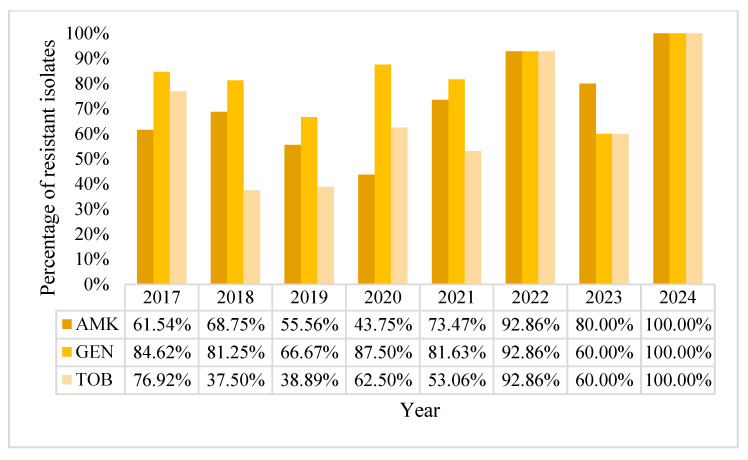
Percentage of *Acinetobacter baumannii* resistance rates to aminoglycosides.

The overall resistance rate in the aminoglycoside group was 67.09% for amikacin, 81.65% for gentamycin, and 59.49% for tobramycin. The tendencies fluctuated over the years without a well-defined pattern but progressed to a 100% resistance rate by 2024 for all three tested aminoglycosides, although the small number of *A. baumannii* isolates from BSI that year was relatively small (n = 6) (Figure 13). Therefore, a definitive pattern of antimicrobial resistance cannot be observed.

**Figure 14:**
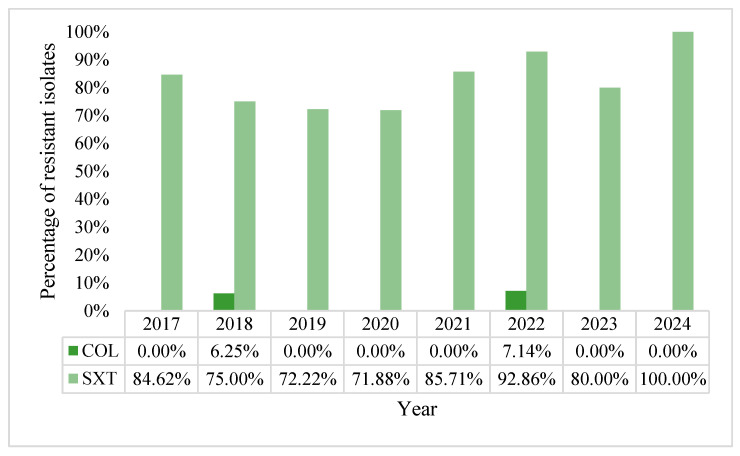
Percentage of colistin and trimethoprim/sulfamethoxazole-resistant strains.

In the group of miscellaneous antibiotics tested, the general resistance rate for colistin was 1.27% and 81.01% for trimethoprim/sulfamethoxazole. The proportion of TMP/SMX-resistant strains remained very high throughout the eight years of the study; the lowest rate was 71.88% in 2020 with a peak of 100% in 2024. Colistin resistance was not that prevalent, with a zenith of 7.14% in 2022, the last year in which such strains were observed in our laboratory (Figure 14).

Out of all *A. baumannii* isolates (n = 137), the proportion of MDR strains was 90.73%. The last year in which non-MDR strains of *A. baumannii* were detected was 2021. In the 2022–2024 period, absolutely all strains were MDR. In the pre-pandemic era, the highest rate was observed in 2017 at 92.31%, and the lowest incidence with 72.22% in 2019. The higher incidence of VAP during the pandemic years could be responsible for such high numbers of MDR strains.

## 3. Discussions

The new antibiotic stewardship strategy for BSI was implemented in our facility in March 2023. For *P. aeruginosa*, the first line of treatment is piperacillin/tazobactam, ceftazidime, amikacin, meropenem, ceftazidime/avibactam, or aztreonam (in case of beta-lactam allergy). The second line is intravenous fosfomycin, colistin, ceftolozan/tazobactam, cefiderocol, levofloxacin, and ciprofloxacin.

For *A. baumannii*, the first line of treatment consists of meropenem, imipenem, gentamycin, trimethoprim/sulfamethoxazole, levofloxacin, colistin, tobramycin, amikacin, and imipenem/relebactam. The second line of treatment is composed of ampicillin-sulbactam and tigecycline.

Given the fact that these two bacteria are also isolated from urine cultures in our hospital, a similar strategy was implemented in the treatment of urinary tract infections.

Since *P. aeruginosa* has a remarkable array of antibiotic resistance mechanisms, including multiple chromosomal determinants and complex regulatory pathways involved in intrinsic and adaptative resistance, antimicrobial therapy should always be guided by AST [31].

The preventive efforts have led to a 29% decrease in infection rates since 2013, indicating a positive trend that may continue, provided the antibiotic stewardship remains in order and/or is improved, guided by a better understanding of local epidemiology of antimicrobial resistance [3]. The same tendency showed up in the study’s last 2022 and 2023 years, which reported one of the lowest incidence rates [4].

Before the COVID-19 pandemic, the prevalence of *P. aeruginosa* was on an uptrend; the same trend was seen in its antimicrobial resistance patterns. Better antibiotic stewardship strategies during the pandemic managed to curtail both the incidence and the AMR of *P. aeruginosa*.

Factors governing *P. aeruginosa* lung infection and blood dissemination have been investigated in the modern medical literature. One study reported a significant increase in virulence factor protein levels for *P. aeruginosa* bacteremia compared to isolates from the same patient at initial sites of infection, indicating the existence of a very complex adaptive mechanism when *P. aeruginosa* reaches the bloodstream, possibly governed by epigenetic pathways [32]. The establishment of *P. aeruginosa* bacteremia from other initial sites must be further characterized to evaluate site-specific factors. Additionally, *P. aeruginosa*’s mechanisms for bloodstream fitness have not been addressed as of yet. Unfortunately, our microbiology laboratory does not yet have the adequate means of detecting said virulence factors. Still, it remains a potential way of implementing more rapid and targeted antimicrobial therapies, possibly with higher success rates and lower morbidity and mortality.

Data from the literature regarding the incidence of *P. aeruginosa* bloodstream infections in a pandemic setting are still somewhat scarce, with no definitive trends emerging. A systematic review from 2023 noticed a slight increase in *P. aeruginosa* bacteremia incidence during the SARS-CoV-2 pandemic [33]. On the contrary, we have noticed a reduction in cases during the pandemic years, possibly due to the implementation of more aggressive antibiotic stewardship programs. Other studies noticed an increase in antibiotic resistance of *P. aeruginosa*, highlighting the need for more sustained efforts to minimize the likelihood of acquiring such infections [34]. We have not noticed a significant increase in antibiotic resistance of *P. aeruginosa* during the years comprised in the study. The emergence of colistin-resistant strains in the post-pandemic years better puts into perspective the need for the development of new salvage therapies for this infection.

Regarding *A. baumannii* bacteremia, our results were worrying, especially if viewed from the lens of a pandemic that stretched thin the resources and resilience of medical personnel. The increase in incidence, coupled with the increase in AMR, paints a more pessimistic image for future pandemics.

A systematic review and meta-analysis from Italy identified previous antimicrobial therapy, central-line catheter use and mechanical ventilation as significant risk factors for developing *A. baumannii* bacteremia and a higher incidence of such infections in intensive care units, while also highlighting the need for implementing thorough infection prevention and control measures (especially in the management of central-line catheters and mechanical ventilation) and antimicrobial stewardship [35]. A retrospective study from Thailand noticed an increase in incidence of carbapenem-resistant *A. baumannii* BSI during the pandemic. It did not, however, find a correlation between carbapenem-resistance and hospital-wide carbapenem use, suggesting that infection control measures may impact curtailment of *A. baumannii* more than carbapenem regulation [36]. Our results were consistent with these findings, with an increase in both incidence and antimicrobial resistance during pandemic years.

Infection prevention strategies implemented in our clinic have reduced *A. baumannii* bacteremia, especially in post-pandemic years, indicating that continued efforts could reduce the disease burden [18].

Being a monocentric study, the findings may reflect the local epidemiology and resistance patterns, somewhat limiting extrapolation to national or international settings. Another limitation of the study is the retrospective nature of it, limiting the ability to control for potential confounders, and it thus relies on the accuracy of existing laboratory and hospital records. Also, the lack of granular and objective clinical data, such as severity of illness scores, previous antibiotic exposure information, and outcome measures (e.g., mortality, length of stay), precluded more robust analyses of prognostic factors.

We were unable to identify the sources of bloodstream infections, such as catheter-associated or ventilator-associated origins, which may have painted a clearer picture of the antimicrobial resistance profiles.

Lastly, some yearly subgroups (such as 2022) had a limited number of isolates, possibly impacting the interpretation of antimicrobial resistance trends. Such data should, therefore, be interpreted cautiously.

## 4. Materials and Methods

### 4.1. Study Design and Setting

This is a retrospective, observational, monocentric study conducted over eight years (January 2017–December 2024) at the National Institute of Infectious Diseases “Prof. Dr. Matei Balș”, Bucharest, Romania. We included all blood cultures positive for *P. aeruginosa* or *A. baumannii* from hospitalized patients between 2017 and 2024 and excluded blood cultures with polymicrobial growth not attributable to either pathogen, or samples considered contaminants based on clinical context. Limited demographic data was collected for each positive blood culture which met the criteria of inclusion in our study.

### 4.2. Blood Culture Processing and Bacterial Identification

Blood samples were collected using BACT/ALERT hemoculture sets (bioMérieux, Salt Lake City, UT, USA) and were processed in the BACT/ALERT 3D automated system and incubated at 35 ± 2 °C for up to five days. Samples which showed bacterial growth were Gram-stained and subcultured on appropriate solid media and incubated aerobically and anaerobically. Plates were examined according to laboratory procedures every 24 h. Species identification was performed using VITEK2 Compact (bioMérieux, Salt Lake City, UT, USA) and matrix-assisted laser desorption/ionization time-of-flight mass spectrometry (MALDI-TOF MS, Bruker, Billerica, MA, USA), with identification considered valid for log(score) ≥ 2.0.

### 4.3. Antimicrobial Susceptibility Testing

Antimicrobial susceptibility testing (AST) was performed using the VITEK2 Compact (bioMérieux, Salt Lake City, UT, USA) and MICRONAUT-AM (Bruker, Billerica, MA, USA) automated systems. Detection of extended-spectrum β-lactamase (ESBL) production and carbapenemase activity was carried out using NG-Biotech immunochromatographic assays (Guipry, France), with confirmation performed using ROSCO Diagnostica kits (Taastrup, Denmark). The AST panel for *P. aeruginosa* contained 11 antibiotics: piperacillin-tazobactam (TZP), ticarcillin-clavulanic acid (TCC), cefepime (CFPM), ceftazidime (CAZ), imipenem (IPM), meropenem (MER), aztreonam (AZT), ciprofloxacin (CIP), levofloxacin (LEV), amikacin (AMK), tobramycin (TOB), and colistin (COL). For *A. baumannii*, the AST panel consisted of 9 antibiotics: imipenem (IMI), meropenem (MER), ciprofloxacin (CIP), levofloxacin (LEV), amikacin (AMK), gentamicin (GEN), tobramycin (TOB), colistin (COL), and trimethoprim-sulfamethoxazole (SXT). VITEK2 MIC outputs were interpreted with the VITEK 2 Advanced Expert System (AES) using the EUCAST clinical breakpoints valid for each study year (versions 2017–2024); any AES ‘Expert Rule’ overrides or discordant results were verified manually against the corresponding EUCAST tables before final reporting [32]. For colistin susceptibility, broth microdilution methods were employed, following EUCAST recommendations. Multidrug resistance (MDR) was defined as non-susceptibility to at least one agent in three or more antimicrobial categories, in line with international consensus criteria.

### 4.4. Statistical Analysis

Demographic and laboratory data were extracted from laboratory records. Descriptive statistics were performed using Microsoft Excel 2019 (Microsoft Corporation, Redmond, WA, USA). Categorical variables were summarized as counts and percentages.

## 5. Conclusions

This eight-year study highlights the significant burden of bloodstream infections caused by *P. aeruginosa* and *A. baumannii* in a Romanian tertiary care hospital. *A. baumannii* showed consistently high multidrug resistance (MDR), reaching 100% in recent years. In contrast, *P. aeruginosa* displayed fluctuating resistance patterns, with temporary improvements during the COVID-19 pandemic likely due to enhanced stewardship, followed by a resurgence. Male patients were more frequently affected by both pathogens.

In an era of increasing antimicrobial resistance, differentiating between colonization and true bacteremia remains essential to avoid overtreatment. Our data support the implementation of targeted, evidence-based strategies that consider local resistance trends to improve patient outcomes and curb the spread of resistant organisms.

## Data Availability

Dataset available on request from the authors.

## References

[1] Murray C.J.L., Ikuta K.S., Sharara F., Swetschinski L., Robles A.G., Gray A., Han C., Bisignano C., Rao P., Wool E. (2022). Global burden of bacterial antimicrobial resistance in 2019: A systematic analysis. Lancet.

[2] WHO (2024). WHO Bacterial Priority Pathogens List, 2024: Bacterial Pathogens of Public Health Importance to Guide Research, Development and Strategies to Prevent and Control Antimicrobial Resistance.

[3] Shoaib M., Tang M., Aqib A.I., Zhang X., Wu Z., Wen Y., Hou X., Xu J., Hao R., Wang S. (2024). Dairy farm waste: A potential reservoir of diverse antibiotic resistance and virulence genes in aminoglycoside- and beta-lactam-resistant Escherichia coli in Gansu Province, China. Environ. Res..

[4] Kim D.W., Cha C.J. (2021). Antibiotic Resistome from the One-Health Perspective: Understanding and Controlling Antimicrobial Resistance Transmission. Experimental and Molecular Medicine.

[5] WHO (2024). Global Priority List of Antibiotic-Resistant Bacteria to Guide Research, Discovery, and Development of New Antibiotics.

[6] Wisplinghoff H., Bischoff T., Tallent S.M., Seifert H., Wenzel R.P., Edmond M.B. (2024). Nosocomial bloodstream infections in US hospitals: Analysis of 24,179 cases from a prospective nationwide surveillance study. Clin. Infect. Dis..

[7] Diekema D.J., Hsueh P.R., Mendes R.E., Pfaller M.A., Rolston K.V., Sader H.S., Jones R.N. (2019). The microbiology of bloodstream infection: 20-year trends from the SENTRY Antimicrobial Surveillance Program. Antimicrob. Agents Chemother..

[8] Marra A.R., Camargo L.F., Pignatari A.C., Sukiennik T., Behar P.R., Medeiros E.A., Ribeiro J., Girão E., Correa L., Guerra C. (2011). Nosocomial bloodstream infections in Brazilian hospitals: Analysis of 2563 cases from a prospective nationwide surveillance study. J. Clin. Microbiol..

[9] Tam V.H., Rogers C.A., Chang K.T., Weston J.S., Caeiro J.P., Garey K.W. (2010). Impact of multidrug-resistant Pseudomonas aeruginosa bacteremia on patient outcomes. Antimicrob. Agents Chemother..

[10] El-Solh A.A., Hattemer A., Hauser A.R., Alhajhusain A., Vora H. (2012). Clinical outcomes of type III Pseudomonas aeruginosa bacteremia. Crit. Care Med..

[11] Gonçalves I.R., Dantas R.C.C., Ferreira M.L., Batistão D.W.D.F., Gontijo-Filho P.P., Ribas R.M. (2017). Carbapenem-resistant Pseudomonas aeruginosa: Association with virulence genes and biofilm formation. Braz. J. Microbiol..

[12] Recio R., Villa J., Viedma E., Orellana M., Lora-Tamayo J., Chaves F. (2018). Bacteraemia due to extensively drug-resistant Pseudomonas aeruginosa sequence type 235 high-risk clone: Facing the perfect storm. Int. J. Antimicrob. Agents.

[13] Rutherford V., Yom K., Ozer E.A., Pura O., Hughes A., Murphy K.R., Cudzilo L., Mitchell D., Hauser A.R. (2018). Environmental reservoirs for exoS+ and exoU+ strains of Pseudomonas aeruginosa. Environ. Microbiol. Rep..

[14] Bertrand X., Thouverez M., Talon D., Boillot A., Capellier G., Floriot C., Hélias J. (2001). Endemicity, molecular diversity and colonisation routes of Pseudomonas aeruginosa in intensive care units. Intensive Care Med..

[15] Antibiotic Resistance Threats in the United States. *2019 Antibiotic Resistance Threats Report.* Antimicrobial Resistance. https://www.cdc.gov/antimicrobial-resistance/data-research/threats/index.html.

[16] Antimicrobial Resistance Threats in the United States. *2021–2022 Antimicrobial Resistance*. https://www.cdc.gov/antimicrobial-resistance/data-research/threats/update-2022.html.

[17] Gary P., Deirdre C., Geraldine H., Elmer K., Paul S., Gail W. (2017). Koneman’s Color Atlas and Textbook of Diagnostic Microbiology.

[18] Russell D.L., Uslan D.Z., Rubin Z.A., Grogan T.R., Martin E.M. (2018). Multidrug resistant Acinetobacter baumanii: A 15-year trend analysis. Infect Control Hosp. Epidemiol..

[19] Xu M., Fu Y., Kong H., Chen X., Chen Y., Li L., Yang Q. (2018). Bloodstream infections caused by Klebsiella pneumoniae: Prevalence of blaKPC, virulence factors and their impacts on clinical outcome. BMC Infect. Dis..

[20] Martín-Aspas A., Guerrero-Sánchez F.M., García-Colchero F., Rodríguez-Roca S., Girón-González J.A. (2018). Differential characteristics of Acinetobacter baumannii colonization and infection: Risk factors, clinical picture, and mortality. Infect Drug Resist..

[21] Lee N.Y., Chang T.C., Wu C.J., Chang C.M., Lee H.C., Chen P.L., Lee C.-C., Ko N.-Y., Ko W.-C. (2010). Clinical manifestations, antimicrobial therapy, and prognostic factors of monomicrobial Acinetobacter baumannii complex bacteremia. J. Infect..

[22] Shenoy E.S., Pierce V.M., Sater M.R.A., Pangestu F.K., Herriott I.C., Anahtar M.N., Bramante J.T., Kwon D.S., Hawkins F.R., Suslak D. (2020). Community-acquired in name only: A cluster of carbapenem-resistant Acinetobacter baumannii in a burn intensive care unit and beyond. Infect. Control Hosp. Epidemiol..

[23] Liou M.L., Chen K.H., Yeh H.L., Lai C.Y., Chen C.H. (2017). Persistent nasal carriers of Acinetobacter baumannii in long-term-care facilities. Am. J. Infect. Control..

[24] Byrne-Bailey K.G., Gaze W.H., Kay P., Boxall A.B., Hawkey P.M., Wellington E.M. (2009). Prevalence of sulfonamide resistance genes in bacterial isolates from manured agricultural soils and pig slurry in the United Kingdom. Antimicrob. Agents Chemother..

[25] Sarma P.M., Bhattacharya D., Krishnan S., Lal B. (2004). Assessment of intra-species diversity among strains of Acinetobacter baumannii isolated from sites contaminated with petroleum hydrocarbons. Can. J. Microbiol..

[26] Smith A.R., Vowles M., Horth R.Z., Smith L., Rider L., Wagner J.M., Sangster A., Young E.L., Schuckel H., Stewart J. (2021). Infection control response to an outbreak of OXA-23 carbapenemase-producing carbapenem-resistant Acinetobacter baumannii in a skilled nursing facility in Utah. Am. J. Infect. Control..

[27] Pendleton J.N., Gorman S.P., Gilmore B.F. (2013). Clinical relevance of the ESKAPE pathogens. Expert Rev. Anti-Infect. Ther..

[28] Thom K.A., Johnson J.K., Lee M.S., Harris A.D. (2011). Environmental contamination because of multidrug-resistant *Acinetobacter baumannii* surrounding colonized or infected patients. Am. J. Infect. Control..

[29] Thom K.A., Maragakis L.L., Richards K., Johnson J.K., Roup B., Lawson P., Harris A.D., Fuss E.P., Pass M.A., Blythe D. (2012). Assessing the burden of *Acinetobacter baumannii* in Maryland: A statewide cross-sectional period prevalence survey. Infect. Control Hosp. Epidemiol..

[30] Chen C.-T., Wang Y.-C., Kuo S.-C., Shih F.-H., Chen T.-L., How C.-K., Yang Y.-S., Lee Y.-T. (2018). Community-acquired bloodstream infections caused by *Acinetobacter baumannii*: A matched case-control study. J. Microbiol. Immunol. Infect..

[31] The European Committee on Antimicrobial Susceptibility Testing Breakpoint Tables for Interpretation of MICs and Zone Diameters. Version 14.0, 2024. https://www.eucast.org/clinical_breakpoints/.

[32] Su L.-X., Meng K., Zhang X., Wang H.-J., Yan P., Jia Y.-H., Feng D., Xie L.-X. (2012). Diagnosing ventilator-associated pneumonia in critically ill patients with sepsis. Am. J. Crit. Care.

[33] Pang Z., Raudonis R., Glick B.R., Lin T.J., Cheng Z. (2019). Antibiotic resistance in *Pseudomonas aeruginosa*: Mechanisms and alternative therapeutic strategies. Biotechnol. Adv..

[34] Baudet A., Regad M., Gibot S., Conrath É., Lizon J., Demoré B., Florentin A. (2024). Pseudomonas aeruginosa Infections in Patients with Severe COVID-19 in Intensive Care Units: A Retrospective Study. Antibiotics.

[35] Magrini E., Rando E., Liguoro B., Salvati F., del Vecchio P., Fantoni M., Torti C., Murri R. (2025). Risk factors associated with bloodstream infections caused by Acinetobacter baumannii in hospital settings: A systematic review and meta-analysis. CMI Commun..

[36] Chotiprasitsakul D., Ao-udomsuk K., Santinirand P. (2025). Impact of COVID-19 on epidemiology and mortality risk factors in patients with carbapenem-resistant Acinetobacter baumannii bloodstream infections in a tertiary care hospital in Thailand. J. Glob. Antimicrob. Resist..

